# Supplementary material for: Fiber laser based stimulated Raman photothermal microscopy towards a high-performance and user-friendly chemical imaging platform
Source: Photonix. 2025 Sep 29;6(1):35. doi: 10.1186/s43074-025-00196-1 (PMC12479582; doi:10.1186/s43074-025-00196-1)
Supplement: Supplementary file 1 — Supplementary Material 1. [file 43074_2025_196_MOESM1_ESM.docx]

**Supplementary Information for:**

**Fiber laser based stimulated Raman photothermal microscopy with long working distance optics**

Xiaowei Ge^a,†^, Yifan Zhu^a,†^, Dingcheng Sun^b^, Hongli Ni^a^, Yueming Li^a^, Chinmayee V. Prabhu Dessai^b^, Ji-Xin Cheng ^a,b,c,d^*

^a^Department of Electrical & Computer Engineering, Boston University, Boston,
Massachusetts, USA.

^b^Department of Biomedical Engineering, Boston University, Boston, Massachusetts, USA.

^c^Department of Chemistry, Boston University, Boston, Massachusetts, USA.

^d^Photonics Center, Boston University, Boston, Massachusetts, USA.

^†^These authors have contributed equally to this work

***Corresponding authors**: [jxcheng@bu.edu](mailto:jxcheng@bu.edu)

**This PDF file includes:**

Figs. S1 to S11

Videos S1 to S3

Tables S1

Supplementary Method

**
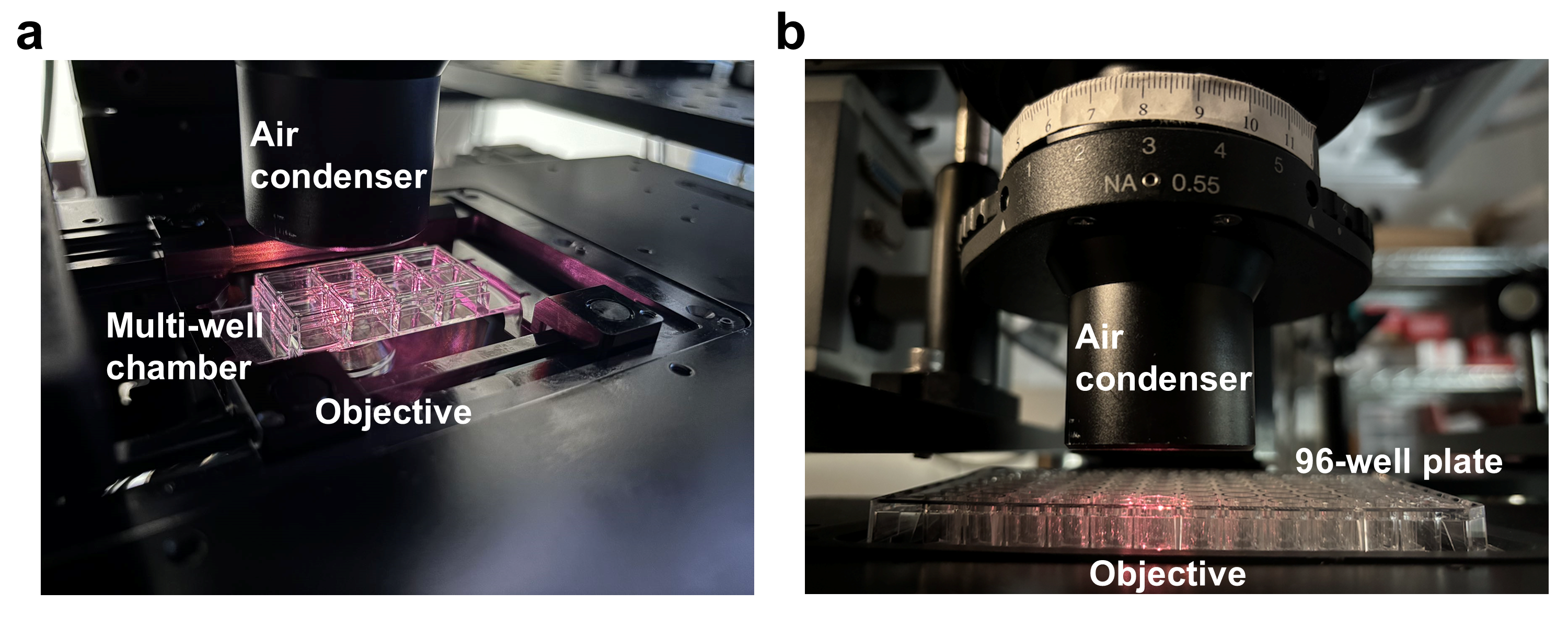
**

**Fig. S1** Experimental setup of SRP. **a**. Long working distance allows the test of multi-well chamber. **b**. Compatibility with 96-well plate.


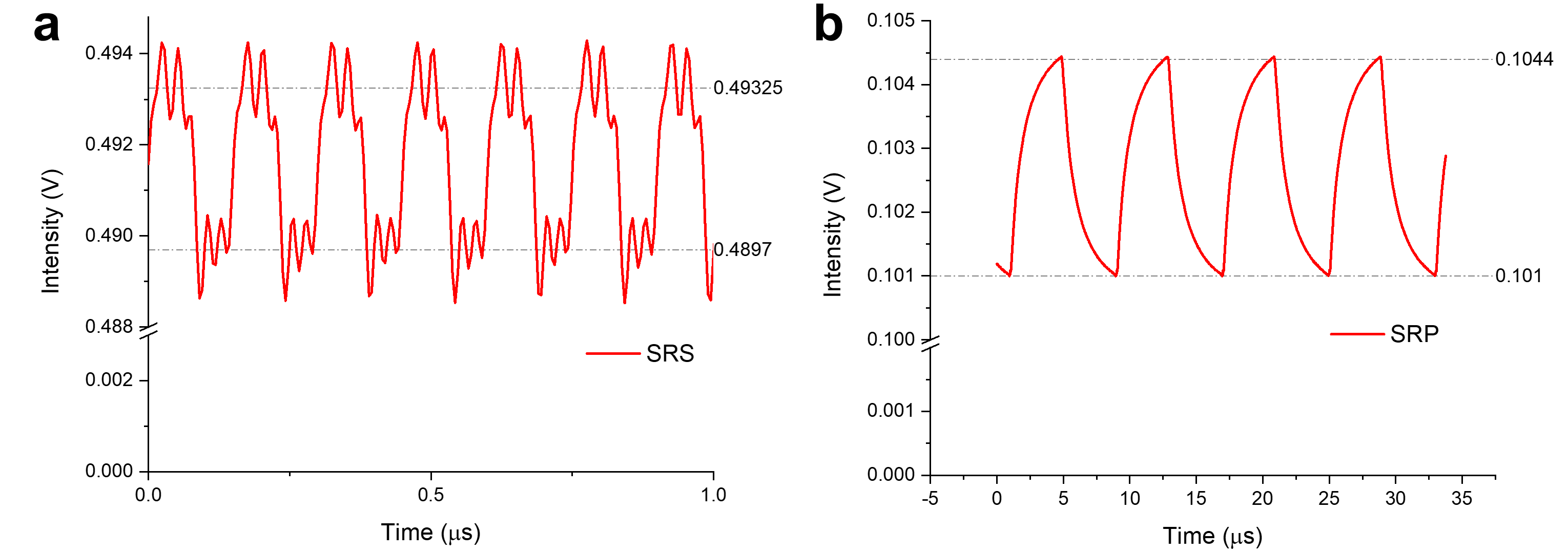


**Fig. S2** Modulation depth compare between SRS and SRP on DMSO. Pump power on sample: 28 mW, no modulation. Stokes power on sample: 90 mW, 50% duty cycle modulation. Probe power on sample: 23 mW. **a**. SRS modulation depth 0.72%. **b**. SRP modulation depth 3.3%.


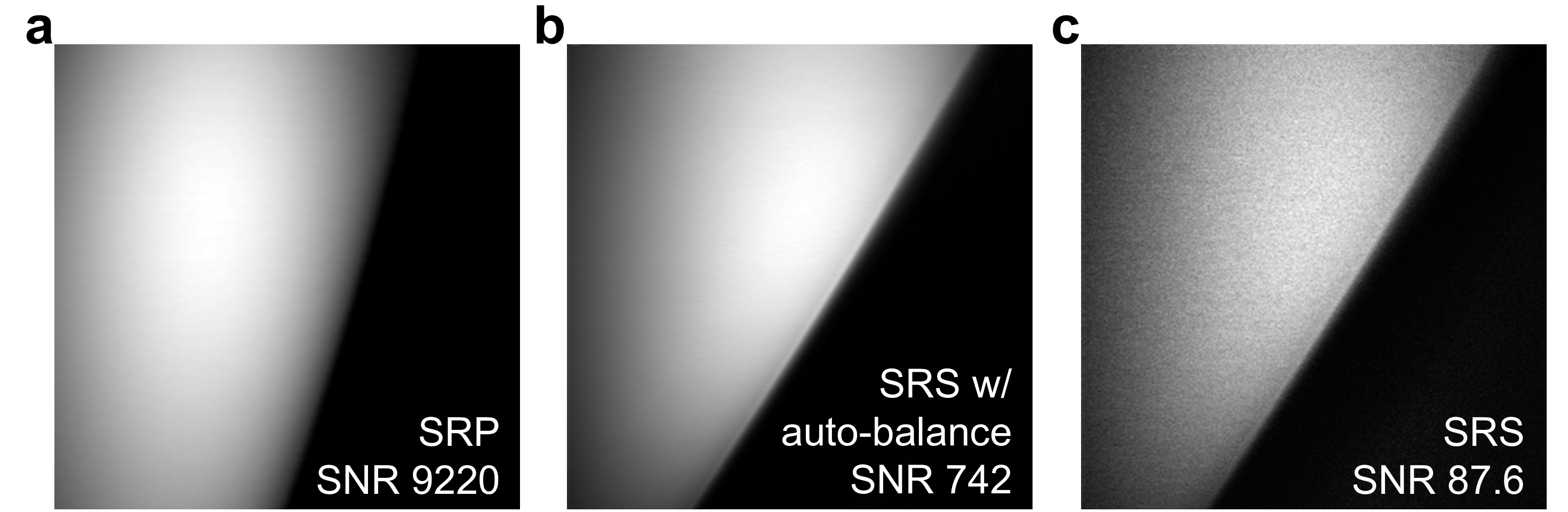


**Fig. S3** SNR comparison of SRP (**a**), SRS with auto-balance detection (**b**) and SRS without balance detection (**c**) on DMSO. Pump power on sample: 28 mW, no modulation. Stokes power on sample: 90 mW, 50% duty cycle modulation. Probe power on sample: 23 mW.


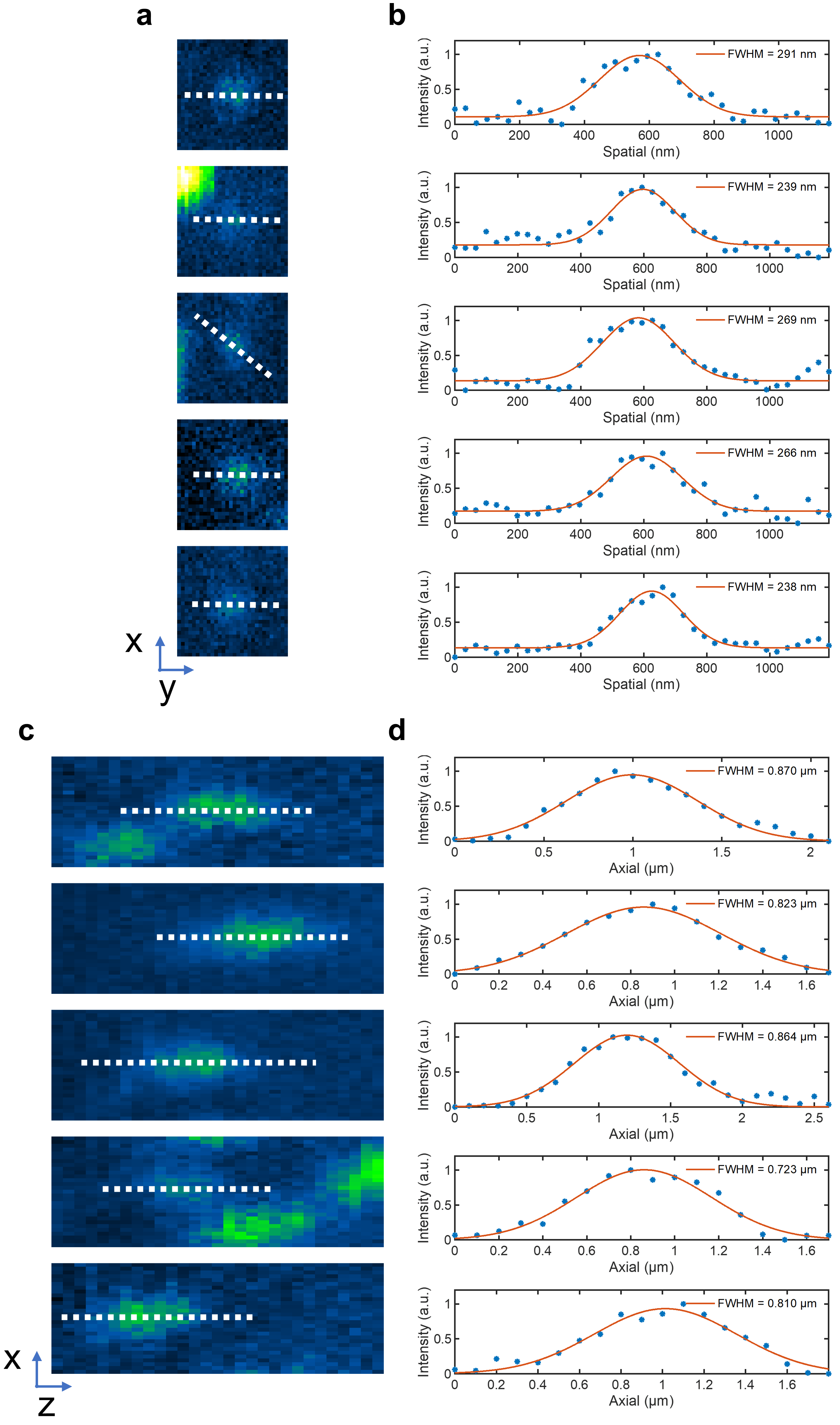


**Fig. S4** SRP spatial resolution characterization. **a**. SRP lateral imaging of 100 nm PMMA beads in glycerol-d8 agar. Scale bar: 300 nm. **b**. Gaussian fitting of profiles by the white dash lines in a. **c**. SRP axial imaging of 100 nm PMMA beads in glycerol-d8 agar. Scale bar: 300 nm. **d**. Gaussian fitting of profiles by the white dash lines in c.


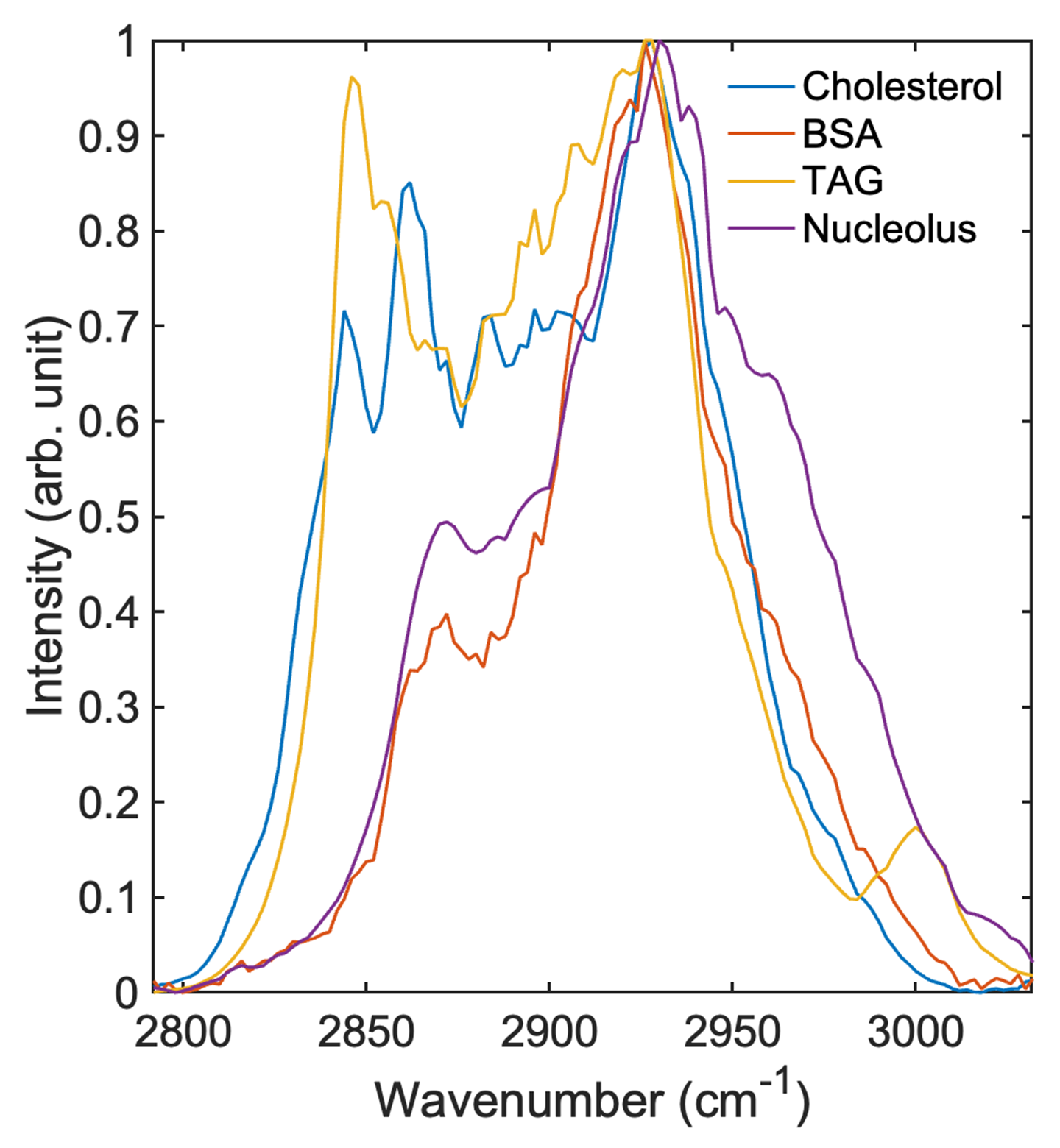


**Fig. S5** LASSO reference from standard sample SRP measurement. BSA: bovine serum albumin. TAG: triacylglycerol.


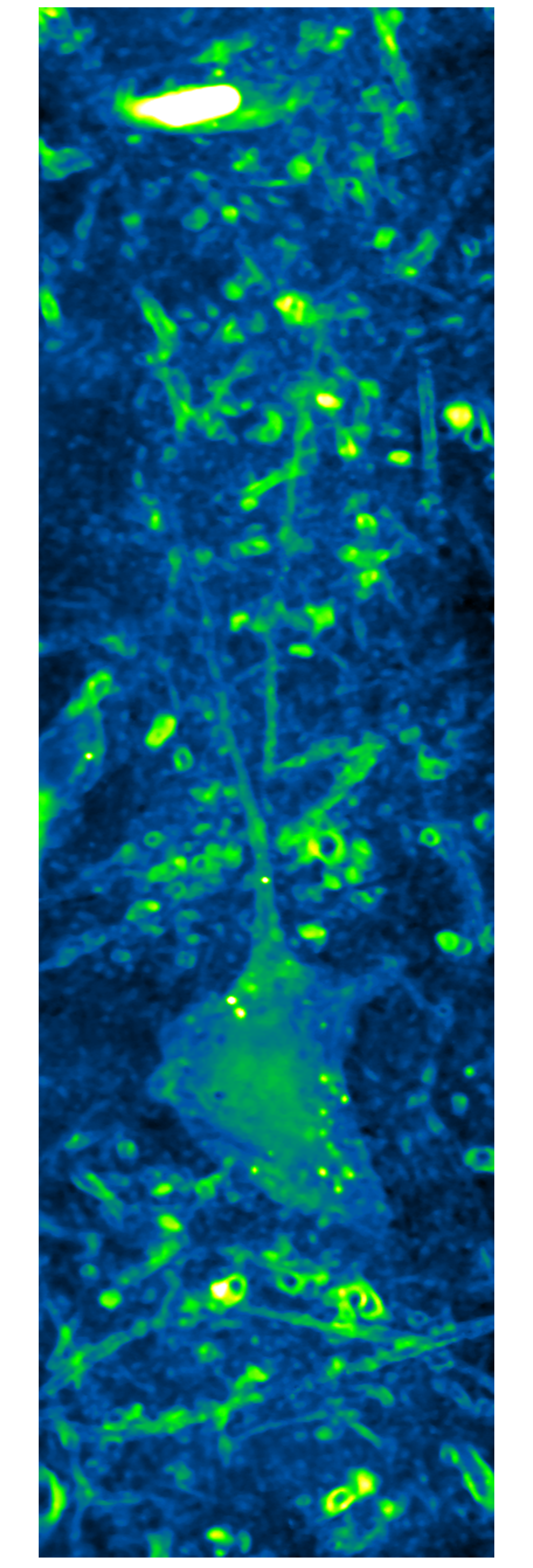


**Fig. S6** Single-color SRP image visualizing a single neuron with its axon and dentrite embedded in a urea-cleared rat brain at 2930 cm^-1^. Scale bar: 10 μm.


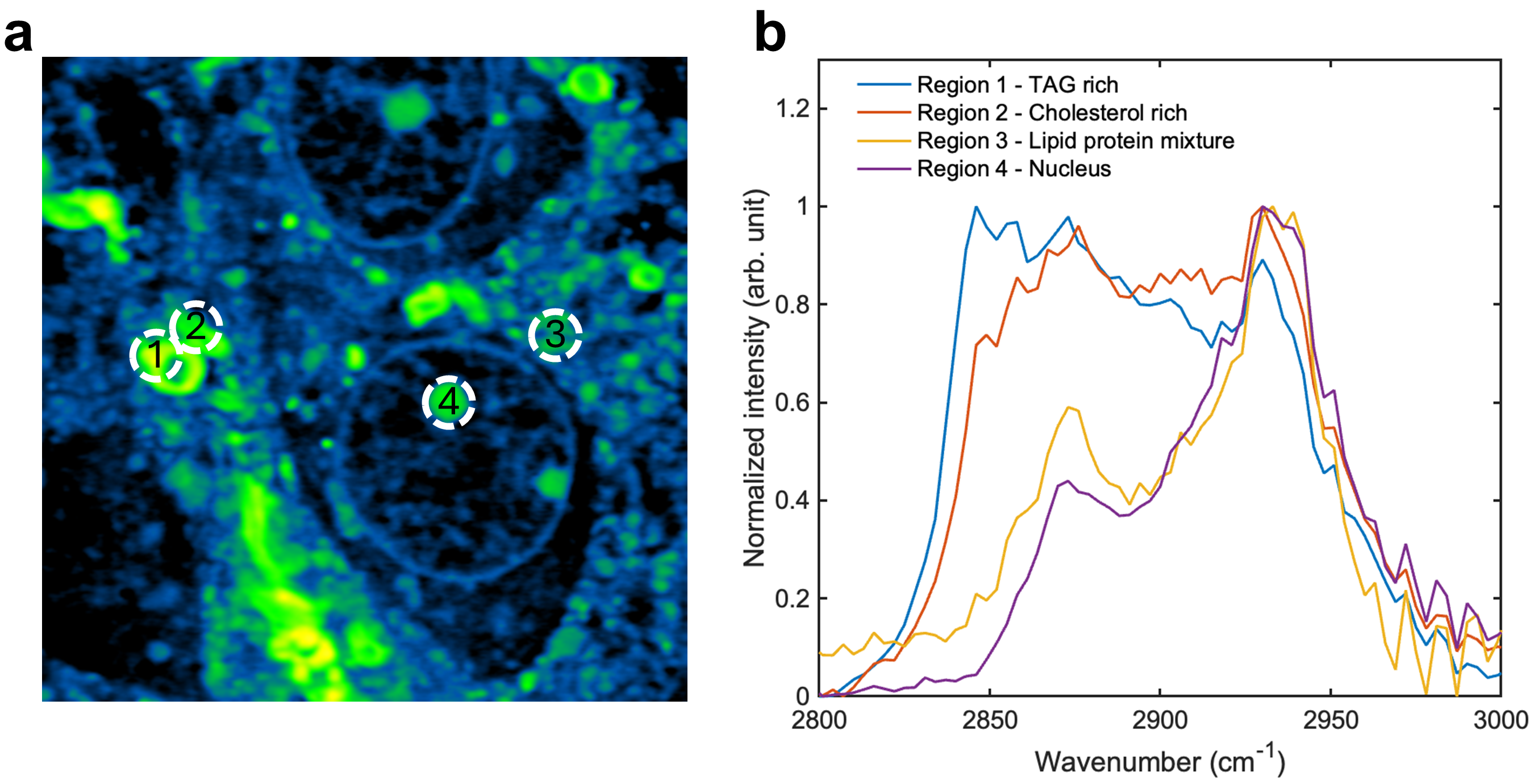


**Fig. S7** Hyperspectral SRP measurement of the tissue clearance rat brain sample. **a**. Single frame of hyperspectral SRP imaging at 2930 cm^-1^. **b**. SRP spectrum of region of interests in a.


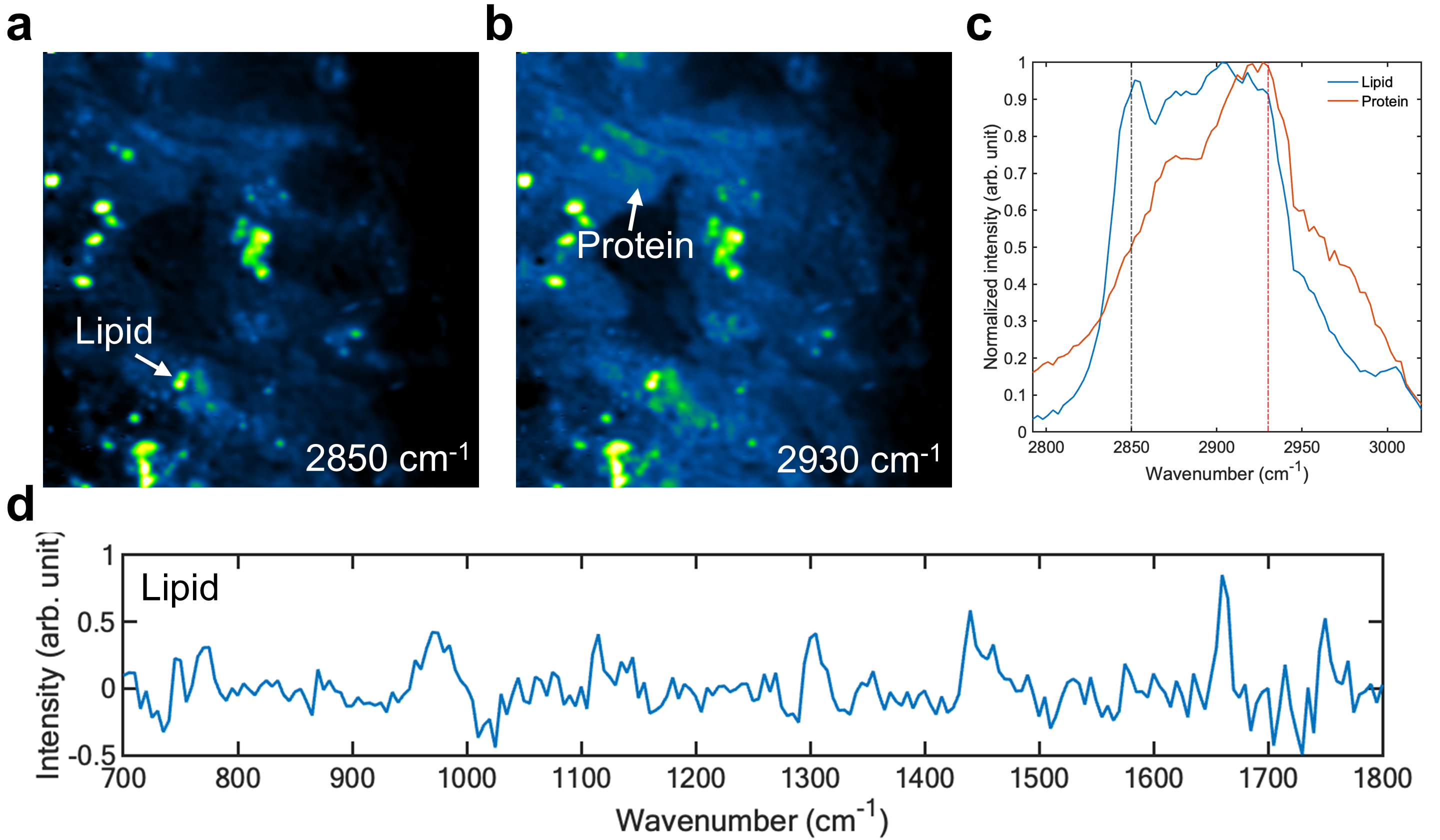


**Fig. S8** Ovarian tumor imaging in whole spectral region. **a.** SRP imaging of glycerol-d8 immersed tumor tissue at 2850 cm^-1^. **b**. SRP imaging of the same FOV in a at 2930 cm^-1^. **c**. C-H region SRP spectrum of lipid and protein pointed in a and b. Gray dashed line at 2850 cm^-1^. Red dashed line at 2930 cm^-1^. **d**. Fingerprint region SRP spectrum of lipid pointed in a.


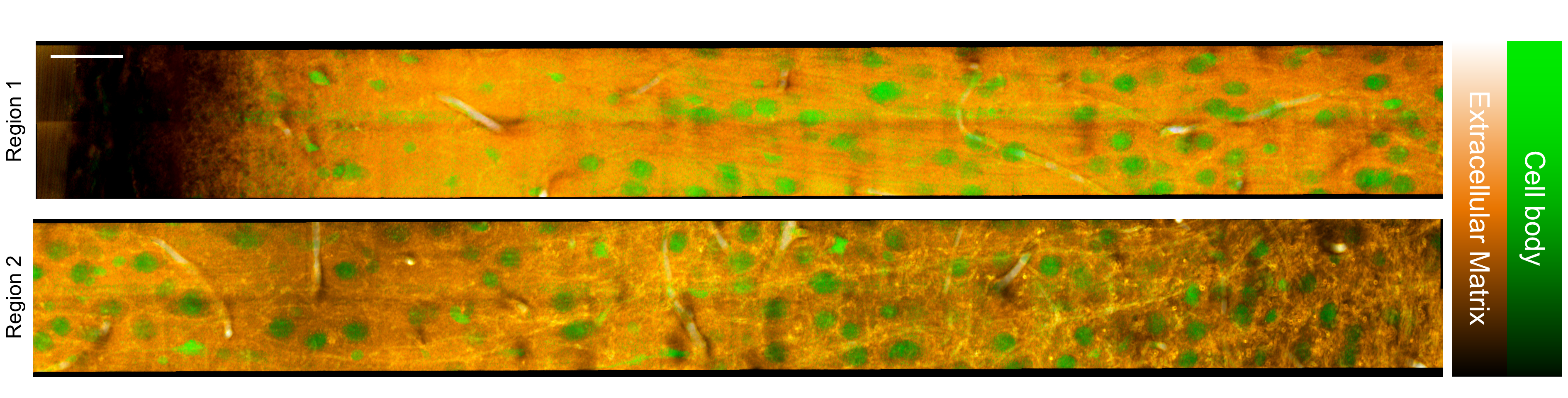


**Fig. S9** 20 μm depth projection of volumetric two-color SRP histology of cerebral cortex. Region 1: V2MM outer region, closer to scalp layer. Region 2: V2MM inner region, close to corpus callosum. Location illustrated in Fig. 7a. Scale bar: 50 μm.


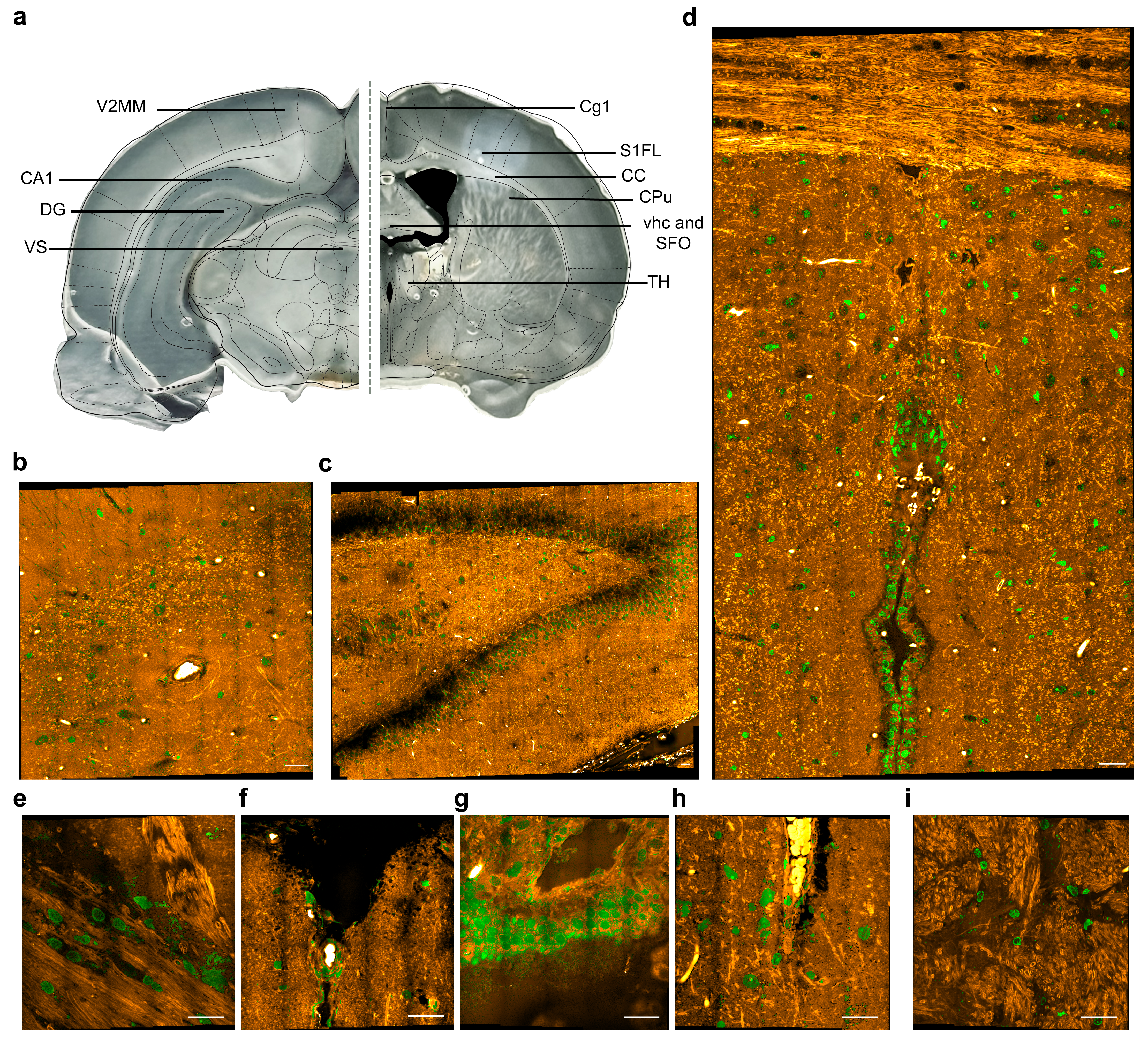


**Fig. S10** Two-color brain histology by fSRP. Scale bar: 30 μm. **a**. Image of the imaged rat brain slices. The left slice is close to the lambda and the right slice is close to the bregma. Two-color SRP histology of cornu ammonis 1 field in hippocampus (CA1) (**b**), dentate gyrus in hippocampus (DG) (**c**), ventricular system (VS) (**d**), primary somatosensory cortex, forelimb region in cerebral cortex (S1FL, top right) and corpus callosum (CC, bottom left) (**e**), cingulate cortex, area 1 in cerebral cortex (Cg1) (**f**), hippocampal commissure (vhc) and subfornical organ (SFO) (**g**), thalamus (TH) (**h**), caudate putamen in cerebral nuclei (CPU) (**i**).


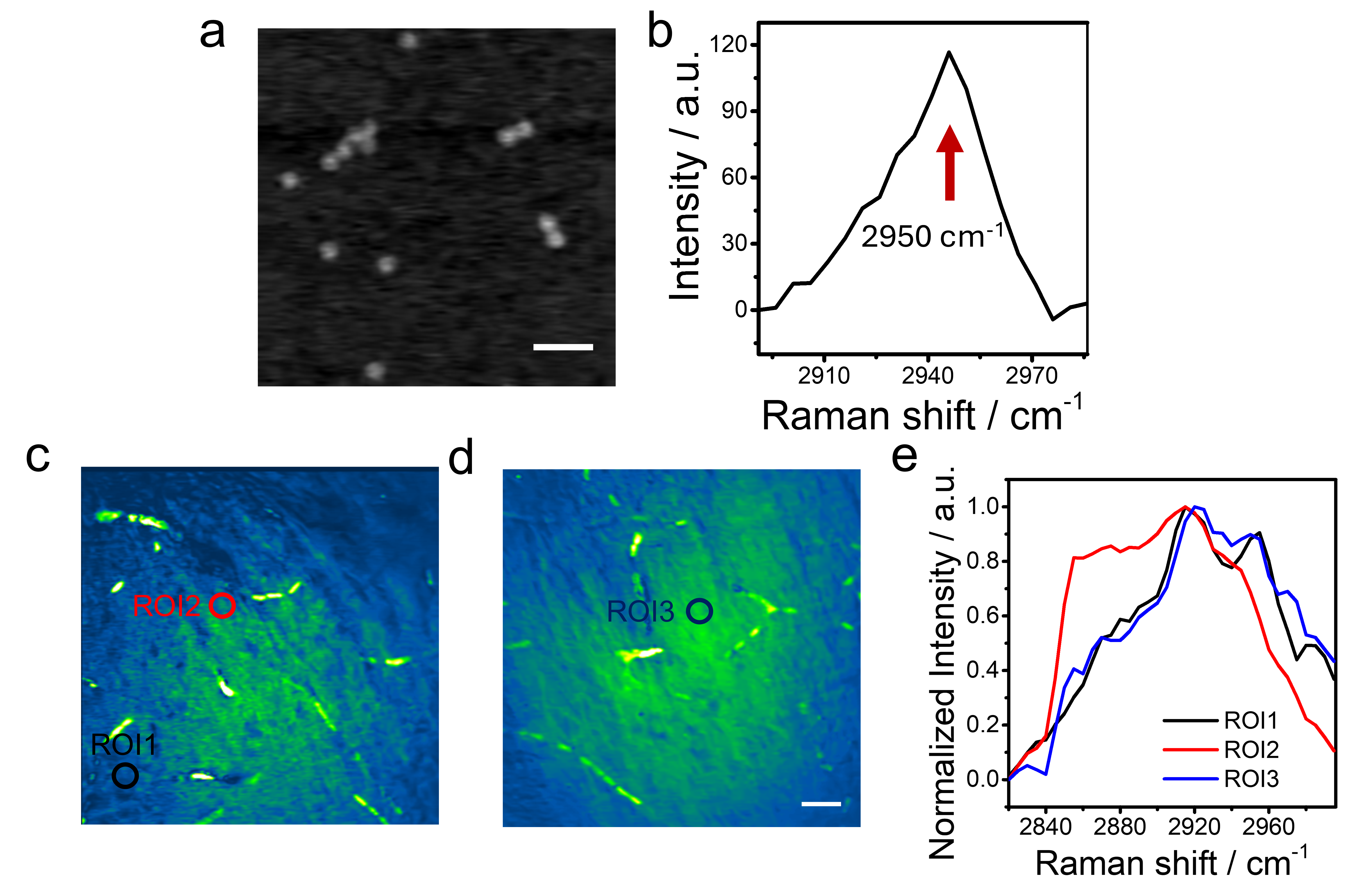


**Fig. S11** Hyperspectral imaging of 3 μm PMMA particles and mouse brain slice by low-NA fSRP. **a**-**b**. low-NA fSRP image (**a**) and spectrum (**b**) of 3 μm PMMA particles. Scale bar: 10 μm. **c**-**d**. low-NA fSRP images of mouse brain slice, at 2930 cm^-1^. Scale bar: 20 μm. **e**. typical SRP spectrum acquired at labeled FOVs.

**Video. S1** Live cell imaging in water condition with lipid movements as shown in Fig. 5g. Plot in logarithmic scale to feature weak features on membrane. Scale bar: 4 μm.

**Video. S2** Single-color depth-resolved SRP imaging of rat brain slice at 2930 cm^-1^. Same FOV as Fig. 7e. Imaging depth: 200 μm. Scale bar: 5 μm.

**Video. S3** Two-color depth-resolved SRP histology of V2MM region (stitching of region 1 and 2 in Fig. 7) in rat cerebral cortex. Imaging depth: 20 μm. Scale bar: 50 μm.

| **Thermal property** | **Unit** | **DMSO** | **glycerol** | **8 M Urea** | **water** |
| --- | --- | --- | --- | --- | --- |
| **Heat Capacity** | **J/(kg·K)** | 1966 | 2400 | 1420 | 4184 |
| **Thermal conductivity** | **W/(m·K)** | 0.200 | 0. 283 | - | 0.598 |
| **Thermo-optic coefficient**  **dn/dT (10^-4^)** | **K^-1^** | -4.93 | -2.30 | - | -1.13 |
| **Refractive index** | **-** | 1.479 | 1.473 | 1.4 | 1.333 |
| **Relative signal intensity** | **a.u.** | 8.37 | 3.21 |  | 1 |
| **Viscosity** | **cP** | 1.991 | - | 1-2 | 0.89 |

**Table S1** Thermal property of the immersion medium used in the study.

**Supplementary Method**

**Preparation of rat brain slice with tissue clearance**

A fresh ovarian tumor was harvested from a euthanized NU/J mouse inoculated with OVCAR5-cisR cells (4-week-old female, homozygous for Foxn1nu, from the Jackson Laboratory). The tumor was immediately fixed in a 10% formalin solution. For cryopreservation, the tissue block was washed with 1× PBS solution (pH 7.4, Thermo Fisher Scientific) and incubated in a 15% sucrose solution for 12 hours. It was then immersed in a 30% sucrose solution overnight at room temperature until the tissue sank. The sample was embedded in optimal cutting temperature (OCT) compound (Fisher Healthcare) and stored at -80°C in a tissue mold prior to sectioning. Tissue slicing was performed using a Leica CM1950 cryostat at the Bio-Interface and Technologies Facility, Boston University. Sections of 10 µm thickness were placed on super frost glass slides and stored at -80°C until imaging. Before imaging, the slides were washed with 1× PBS to remove residual OCT and covered with fresh 1× PBS solution. The sample was then embedded in glycerol-d8 and covered with a glass coverslip for imaging shown in Fig. S8.
