# Supplementary figures and images for: Fiber laser based stimulated Raman photothermal microscopy towards a high-performance and user-friendly chemical imaging platform
Source: Photonix. 2025 Sep 29;6(1):35. doi: 10.1186/s43074-025-00196-1 (PMC12479582; doi:10.1186/s43074-025-00196-1)

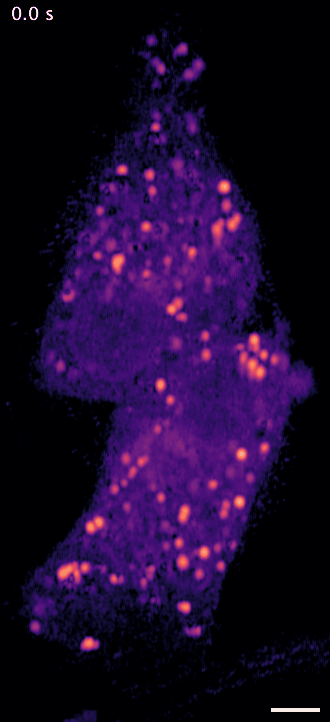

Supplement: Supplementary file 2 — Supplementary Material 2. [file 43074_2025_196_MOESM2_ESM.gif]

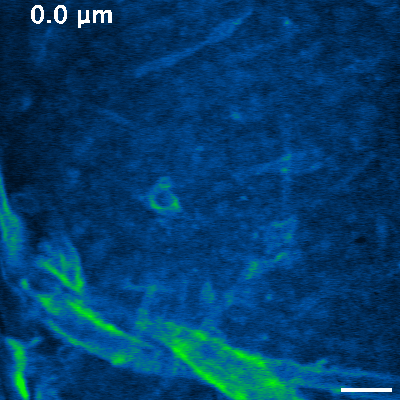

Supplement: Supplementary file 3 — Supplementary Material 3. [file 43074_2025_196_MOESM3_ESM.gif]
